# Isoform Sequencing Provides a More Comprehensive View of the *Panax ginseng* Transcriptome

**DOI:** 10.3390/genes8090228

**Published:** 2017-09-15

**Authors:** Ick-Hyun Jo, Jinsu Lee, Chi Eun Hong, Dong Jin Lee, Wonsil Bae, Sin-Gi Park, Yong Ju Ahn, Young Chang Kim, Jang Uk Kim, Jung Woo Lee, Dong Yun Hyun, Sung-Keun Rhee, Chang Pyo Hong, Kyong Hwan Bang, Hojin Ryu

**Affiliations:** 1 Department of Herbal Crop Research, National Institute of Horticultural and Herbal Science (NIHHS), Rural development administration (RDA), Eumseong 27709, Korea; intron@korea.kr (I.-H.J.); qltbsm@korea.kr (C.E.H.); ycpiano@korea.kr (Y.C.K.); k2korea@korea.kr (J.U.K.); enzymer@korea.kr (J.W.L.); hyundy@korea.kr (D.Y.H.); 2 Department of Biology, Chungbuk National University, Cheongju 28644, Korea; jinsulee90@gmail.com (J.L.); wsbae80@gmail.com (W.B.); 3 TheragenEtex Bio Institute, Suwon 16229, Korea; dongjin.lee@theragenetex.com (D.J.L.); singi.park@theragenetex.com (S.-G.P.); yongju.ahn@theragenetex.com (Y.J.A.); 4 Department of Microbiology, Chungbuk National University, Cheongju 28644, Korea; rhees@chungbuk.ac.kr

**Keywords:** isoform sequencing, *Panax ginsing* C.A. Meyer, transcriptomic profile, genomics

## Abstract

Korean ginseng (*Panax ginseng* C.A. Meyer) has been widely used for medicinal purposes and contains potent plant secondary metabolites, including ginsenosides. To obtain transcriptomic data that offers a more comprehensive view of functional genomics in *P. ginseng*, we generated genome-wide transcriptome data from four different *P. ginseng* tissues using PacBio isoform sequencing (Iso-Seq) technology. A total of 135,317 assembled transcripts were generated with an average length of 3.2 kb and high assembly completeness. Of those unigenes, 67.5% were predicted to be complete full-length (FL) open reading frames (ORFs) and exhibited a high gene annotation rate. Furthermore, we successfully identified unique full-length genes involved in triterpenoid saponin synthesis and plant hormonal signaling pathways, including auxin and cytokinin. Studies on the functional genomics of *P. ginseng* seedlings have confirmed the rapid upregulation of negative feed-back loops by auxin and cytokinin signaling cues. The conserved evolutionary mechanisms in the auxin and cytokinin canonical signaling pathways of *P. ginseng* are more complex than those in *Arabidopsis thaliana*. Our analysis also revealed a more detailed view of transcriptome-wide alternative isoforms for 88 genes. Finally, transposable elements (TEs) were also identified, suggesting transcriptional activity of TEs in *P. ginseng*. In conclusion, our results suggest that long-read, full-length or partial-unigene data with high-quality assemblies are invaluable resources as transcriptomic references in *P. ginseng* and can be used for comparative analyses in closely related medicinal plants.

## 1. Introduction

Korean ginseng (*Panax ginseng* C.A. Meyer) is one of the most important perennial herbs and has been used as a crude drug for thousands of years in Asian countries, especially South Korea, Japan and China [1]. Its effects have been well defined via clinical trials and animal tests and include improved immunity, increased energy, and rejuvenation [2]. The medicinal constituents of ginseng are derived from the roots, in which the active pharmacological compounds gradually increase over long periods of successive cultivation (4–6 years) [3]. Recently, the aerial parts, including ginseng berries, leaves and sprouts, have been discovered as a source of medicinal compounds [4,5,6]. Studies on ginseng have focused on its pharmacological effects on humans [7]. However, functional genomic studies of *P. ginseng* are uncommon due to its long life cycle and the current lack of genomic data [8,9].

Because *P. ginseng* is a perennial allotetraploid plant (2*n* = 4× = 48) with a large genome size (3.2 Gbp) and a considerable amount of repetitive DNA [10,11], it has been challenging to elucidate its genomic structure. Recent advances in sequencing technology have facilitated large-scale transcriptome sequencing, enabling gene expression studies as well as functional genomic studies. In particular, transcriptome profile studies (primarily of roots) in *P. ginseng* and close relatives have provided genomic resources to identify candidate genes associated with traits such as triterpenoid saponin biosynthesis [12,13]. However, transcripts produced with the Illumina Hi-seq platform were short, incomplete and provided limited DNA structural information [14,15]. With genomic information, genomic resources such as molecular markers, genome assembly and transcriptome data can be developed to improve crop breeding and other applications of *P. ginseng* research [16,17].

Recently, single-molecule real-time (SMRT) sequencing, a new method of sequence analysis, was developed and applied to elucidate the genomic structures of difficult-to-sequence organisms [18]. Using this technique, sequences are analyzed from a single strand of DNA without genomic amplification. Compared to prior next-generation sequencing (NGS) techniques, SMRT has the advantage in that it produces long read lengths and is able to analyze trace amounts of DNA. Furthermore, by omitting the DNA amplification step, (which is essential in NGS), sequencing errors associated with the PCR process are prevented, and low-abundance variants can be detected without increasing coverage depth [19]. Recently, studies using SMRT sequencing for the accurate prediction and verification of plant genetic models have recently been conducted, and SMRT sequencing was successfully used for hexaploid wheat [20] and maize [21], both of which have a large genome size and a high percentage of sequence repeats, making assembly particularly difficult. Consequently, a large number of contigs were assembled for each chromosome, thereby increasing the confidence in gene annotation. 

As a strategy to improve the overall accuracy of gene prediction in *P. ginseng*, we generated full-length and/or partially assembled transcript data derived from four different types of *P. ginseng* tissues, including flower, leaf, stem, and root, using a PacBio SMRT sequencing approach with Isoform sequencing (Iso-Seq). We successfully assembled 135,317 unigenes in *P. ginseng* and functionally categorized 120,626 annotated unigenes by using in silico methodology. Our results will allow high-precision gene annotation, will lead to the discovery of novel genes and will be valuable in functional genomic studies in *P. ginseng* and related species.

## 2. Materials and Methods 

### 2.1. Plant Sampling and RNA Preparation

All four tissues, including flowers, leaves, stems and roots, were harvested in the month of June, 2016 from a 4-year-old *P. ginseng* cultivar (Cheonmyeong) growing in the natural environment at the National Institute of Horticultural and Herbal Science (NIHHS) of Rural Development Administration (RDA) in Eumsung (127°45′13.14″ E, 36°56′36.63″ N), Republic of Korea. All tissues were cut into small pieces, frozen by liquid nitrogen, and then extracted using the easy spin RNA extraction kit (iNtRON Biotecnology, Seongnam, Korea) according to the manufacturer’s instructions. The integrity of total RNA was determined using a BIOSPEC-NANO spectrophotometer (Shimadzu, Kyoto, Japan) and agarose gel electrophoresis.

### 2.2. PacBio Iso-Seq

cDNA was size-selected in fractions of lengths 1–2 kb, 2–3 kb, 3–6 kb, and >6 kb from RNAs pooled from four tissues, which contain leaf, stem, root and reproductive organs (fruits and flowers). SMRTbell Template libraries were made from those cDNAs for sequencing on PacBio RS II system as recommended by Pacific Biosciences (Palo Alto, CA, USA). The templates were sequenced via polymerase binding using the DNA polymerase binding kit P6 v2 primers.

### 2.3. Iso-Seq Assembly and Quality Assessment

Iso-Seq assembly was performed using SMRT-Analysis software v2.3.0 [22]. First, Read of Inserts (ROIs) were generated using minimum read quality of 75. The Iso-Seq classify tool classified the ROIs into full-length nonchimeric and non-full length reads. The classification was carried out by identifying 5′ and 3′ adapters used in library preparation. Full-length reads were defined as containing both adapters. Iso-Seq cluster tool was then used for clustering all the full-length reads. In the last step, the consensus sequences produced by the cluster tool were polished using the non-full length reads through the Quiver algorithm [22]. The high and low quiver consensus isoforms were clustered by CD-HIT with a sequence identity threshold of 0.99 [23]. Protein coding sequences (CDSs) were analyzed by using TransDecoder with the following steps [24]; (1) search for all possible CDSs; (2) verify CDSs by GeneID software [25]; and (3) choose the region with the highest score. To assess assembly quality of unigenes, CEGMA [26], which assesses high reliable set of gene annotations in genome and transcriptome assembly, was employed. The compatibility of unigenes to other datasets was analyzed by mapping with BWA [27] using RNA-Seq samples that were derived from 16 tissues of *P. ginseng* reported by Wang et al. (2015) [13], with the following parameters; -k 19 for minimum seed length, -A1 for matching score, -B 4 for mismatch penalty, and -T 30 for alignment output with score higher than 30.

### 2.4. Unigene Annotation

For functional annotation, unigenes were searched against the UniProt, NCBI non-redundant (NR), TAIR, PlantTFDB databases using BLASTX [10] with an *E*-cutoff value of 10^−5^. Protein domains were also searched using InterProScan [28]. Gene ontology (GO) and Kyoto Encyclopedia of Genes and Genomes (KEGG) pathway annotations were performed using Blast2GO [29]. Transposable elements (TEs), microsatellites, and other repeats were screened using RepeatMasker, which was developed for de novo repeat family identification and modeling [30]. In particular, a TE sequence database was constructed from the genomic sequences of *Panax notoginseng* [31] by searching for known TEs coding sequences against Repbase [32] using TBLASTX with a cutoff 10^−10^. The TE sequences searched were annotated with reference of Gydb [33]. SSRs (simple sequence repeats) were searched using SSR finder [34].

### 2.5. Identification of Alternative Splicing Isoforms

To identify alternative splicing events of internal exon(s) that corresponded to exon skipping, we used BLASTN to search for unigenes that showed exact matches for the 100 bases at the 5′ and 3′ ends within their complete open reading frames (ORFs). Then, sequence identities among the unigenes were examined with reciprocal BLAST. The unigenes were clustered, and those that exhibited insertions/deletions of more than 100 bases were selected to exclude ambiguous alternative splicing isoforms, such as heterogeneous forms with almost identical sequences. Finally, the resulting clusters were validated by CLUSTALW [35].

### 2.6. Plant Hormone Treatments and Real Time qRT-PCR

One-year-old ginseng seedlings were transferred to 1/2 B5 liquid medium with or without plant hormones (auxin, NAA 10 μM, Sigma # n0640 and cytokinin, *t*-zeatin 1 μM, Sigma # z0876) and incubated for 2 h. After treatment, the samples were immediately frozen in liquid nitrogen. To extract total RNA, the Easy-Spin IIp Plant RNA Extraction Kit (iNtRON Biotecnology, Seongnam, Korea) was used. cDNA was synthesized with a first-strand synthesis KIT (Enzynomics, Daejeon, Korea). One microgram of total RNA was used. The reaction conditions were as follows: 50 °C for 60 min, 95 °C for 5 min, and 1 min of cooling on ice during the synthesis of first strand cDNA. Selected target genes were subjected to quantitative real-time PCR under the same reaction conditions. The primers were designed using Primer Express 3.0.1 software (Table S6, PE Applied Biosystems, Foster City, CA, USA). qRT-PCR analysis was performed using the QuantBase 3 (Applied Biosystem, Foster City, CA, USA) instrument with SYBR Green Real-time PCR Master Mix (Applied Biosystem, Foster City, CA, USA) according to the manufacturer’s recommendations. The primer sequences used in this study are listed in Table S5. The expression levels using the threshold cycle (Ct) value were calculated using the 2^−ΔΔCt^ method. 

### 2.7. GenBank Accession Code 

The PacBio sequence data generated for this work are accessible via the NCBI Sequence Read Archive under accession number (SRA: SUB2796783).

## 3. Results

### 3.1. Transcriptome Sequencing of P. ginseng Using PacBio Iso-Seq 

**Figure 1:**
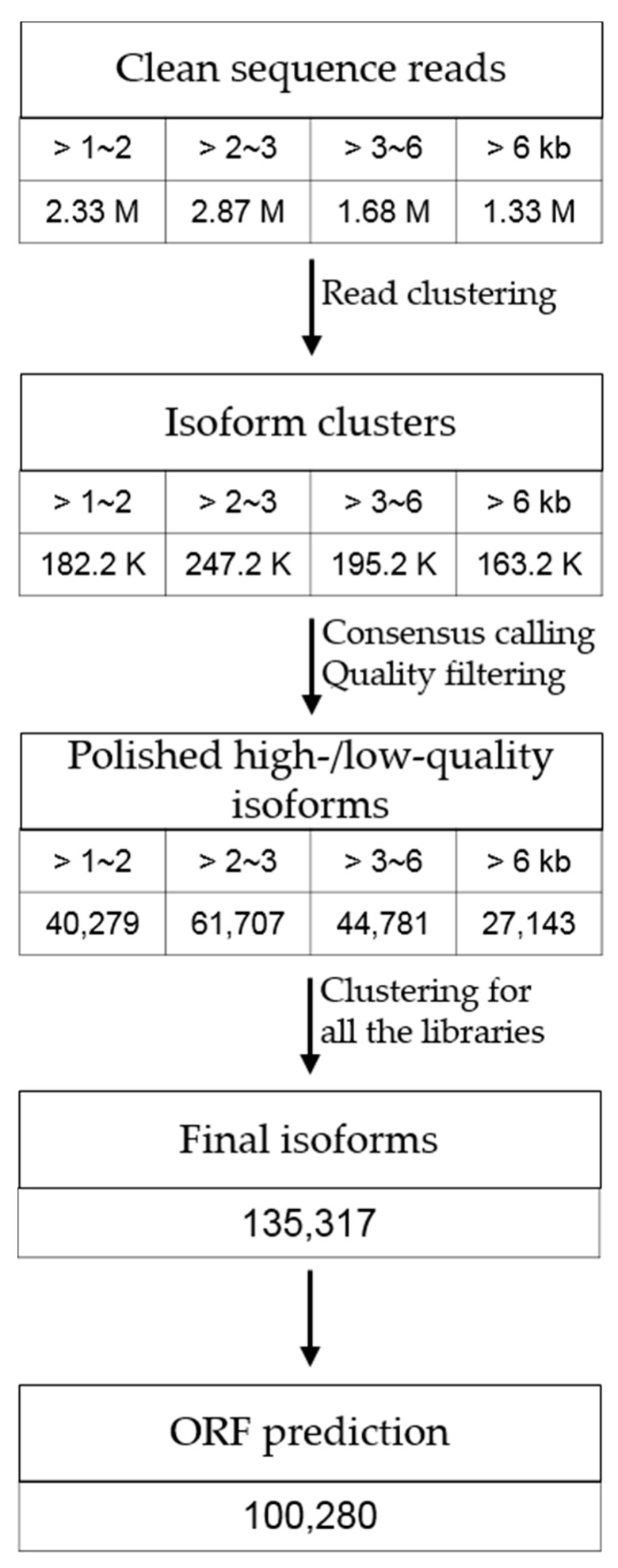
Procedure of transcript assembly of *Panax ginseng* using Iso-Seq. Sub-reads were merged from 163,195 to 247,189 isoform clusters and classified as full-length (FL) and/or non-FL reads. After consensus sequence calling and quality filtration were performed, sequences were finally clustered into 135,317 isoforms. Open reading frames (ORFs) were also predicted in those unigenes.

We generated large *P. ginseng* cDNA fragments using the PacBio Iso-Seq, which favors the reverse transcription of intact, full-length RNA molecules and identifies splice variants of genes. To obtain equal samples of long and short transcripts, the cDNA was size-selected in lengths of 1–2 kb, 2–3 kb, 3–6 kb, and >6 kb from RNA pooled from four tissues, including leaves, stems, roots and reproductive organs (flowers). Using SMRT sequencing technology, a total of 8.2 million sequencing subreads were generated (Table S1) and merged, from 163,195 to 247,189 isoform clusters that were classified as full-length and non-full-length reads (Table S2). After consensus sequence calling and quality filtration (Table S3), in which the sequences were well distributed in the size fractions of libraries (Figure S1), the sequences were clustered into a total of 135,317 isoforms with 90% coverage and 99% sequence identity among sequence overlaps and 91.94% assembly completeness (Figure S2), accounting for approximately 430 Mb in cumulative length. This procedure is summarized in Figure 1. The average length of clustered transcripts (unigenes) was 3178 bp with a length distribution of 2–4 kb (Figure S3), indicating that our dataset contained larger unigenes compared with previous datasets, in which unigenes ranged from 0.79 kb to 1.9 kb [9,13,36]. ORFs were found in 100,280 unigenes (74.1%), with an average length of 756 bp and a cumulative length of approximately 76.9 Mb. Remarkably, 91,345 unigenes were predicted as complete full-length ORFs.

**Figure 2:**
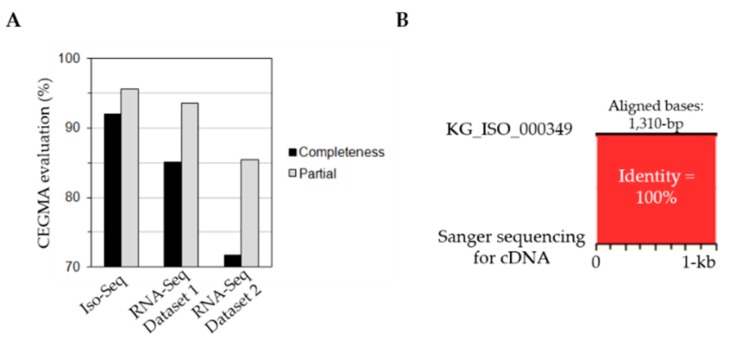

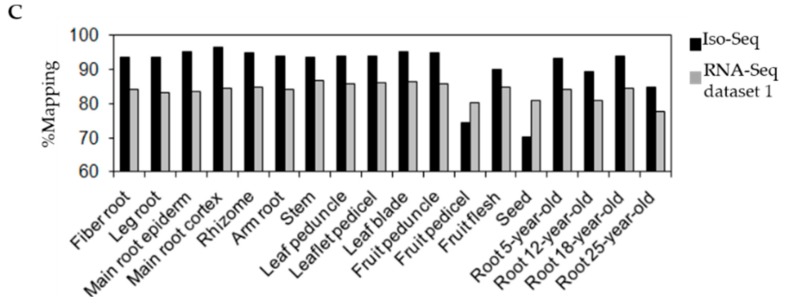
Performance assessment of *P. ginseng* Iso-Seq. (**A**) CEGMA evaluation. The completeness (%) of de novo assemblies from Iso-Seq and RNA-Seq data were assessed by CEGMA. In the result, RNA-Seq dataset 1 and dataset 2 unigenes were generated from data by Wang et al. (2016) [13] and from our unpublished leaf RNA-Seq data, respectively. (**B**) Validation of an unigene by Sanger sequencing. Sequence identity between unigene KG_ISO_000349 and the corresponding Sanger sequence was examined using zPicture. (**C**) Performance assessment of *P. ginseng* Iso-Seq. Mapping of RNA-seq dataset 1, derived from 16 tissues, to the two unigene sets assembled from Iso-Seq and RNA-Seq dataset 1 of *P. ginseng* using BWA.

We also assessed the accuracy and performance of the *P. ginseng* unigenes. The CEGMA evaluation, which assesses a highly reliable set of gene annotations in genome and transcriptome assembly, revealed a very complete (91.94%) dataset compared with the two de novo assembly results, dataset 1 (85.08%) and dataset 2 (71.77%), which were generated using RNA-Seq. These RNA-Seq samples, dataset 1 and dataset 2, were derived from 16 tissues reported by Wang et al. (2015) dataset 1 [13] and produced from *P. ginseng* leaves (dataset 2, unpublished), respectively (Figure 2A). To assess the quality and novelty of our sequencing data, we compared the transcript lengths between our PacBio data and the Illumina RNA-seq de novo assembled unigenes. As presented in Figure S4, almost 56%, 24% and 12% of unigenes distributed in <1 kb, 1–2 kb and 2–3 kb, respectively, were unaligned with the previously reported unigene dataset 1 ([13], Figure S4). However, the PacBio data could recover many portions of longer transcripts than those analyzed using the Illumina RNA-seq de novo assembly (Figure S5). Next, exact matches of the unigenes were validated by Sanger sequencing (i.e., 100% sequence identity in KG_ISO_000349) (Figure 2B). Finally, the RNA-Seq samples derived from the 16 tissues of *P. ginseng* reported by Wang et al. (2015) [13] were mapped to unigenes generated by Iso-Seq (Figure 2C) with an average mapping rate of 90.8%, although they were poorly mapped due to the absence of fruit pedicel (74.4%) and seed (70.1%) tissue in the pooled RNA samples. Mapping rate of the RNA-seq samples to RNA-seq dataset 1 was 83.8% on average. The high mapping rate for Iso-Seq was also supported by a comparison of sequence alignment using STAR which showed an average mapping rate of 83.4% for Iso-Seq and 66.3% for RNA-Seq dataset 1, respectively (Figure S6). Our results suggest that high-quality, longer full-length sequencing reads are an invaluable resource and a reliable transcriptome reference in *P. ginseng*.

### 3.2. Identification of Repeat Sequences in P. ginseng Unigenes

**Figure 3:**
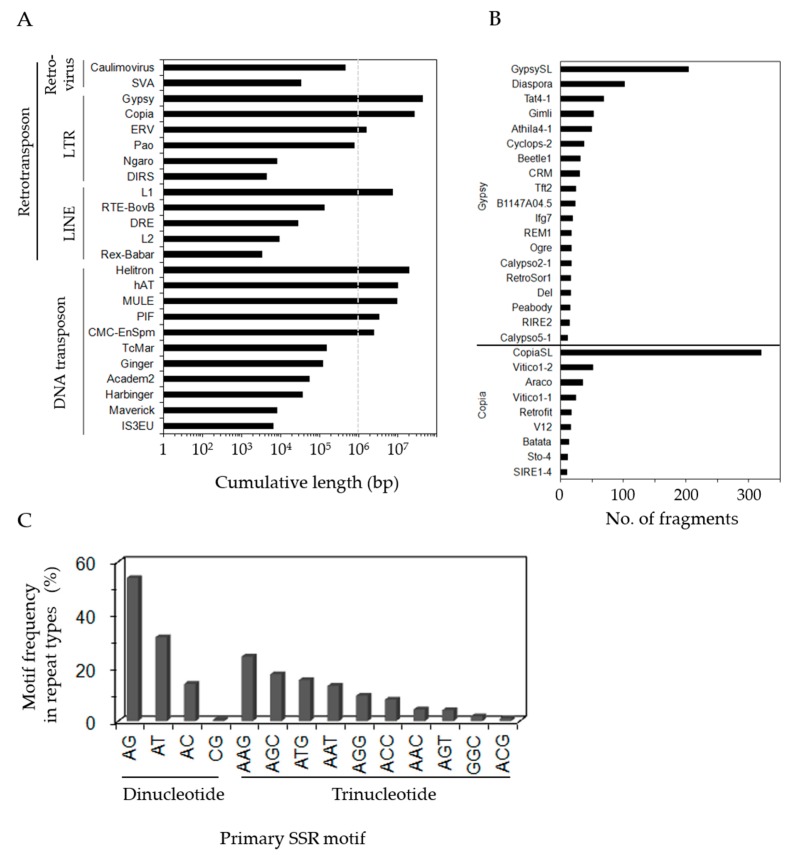
The abundance of repeat sequences in unigenes. (**A**) The distribution of transposable element (TE) sequences in the unigenes. Identified TE sequences were classified into class I (retrotransposons) and class II (DNA transposons) elements with more detailed families. (**B**) The distribution of *Ty3/gypsy*- and *Ty1/copia*-encoding sequences annotated from Gydb. (**C**) Frequency of simple sequence repeat (SSR) motifs discovered in the unigenes from *P. ginseng* based on representing various repeat-motif types. LTR: long terminal repeat; LINE: long interspersed nuclear element.

We next identified an abundance of TE sequences in unigenes by searching against a TE-related coding sequence database constructed from the genome of *P. notoginseng*. These TE sequences accounted for approximately 17.9 Mb (4.2%) in cumulative length. Of TEs searched, long terminal repeat (LTR) retrotranspons (58.2% out of the searched TE sequences, Figure S7), especially *Ty3/gypsy* (34.9%) and *Ty1/copia* (21.4%), were predominantly distributed (Figure 3A). The analysis of *Ty3/gypsy*- and *Ty1/copia*-encoding sequences including ORFs for *gag*, *pol* and other accessory genes revealed the abundance of non-chromodomain retrotransposons for *Ty3/gypsy* and LTR retrotransposons including *copia*-like hemiviruses and sireviruses for *Ty1/copia* (Figure 3B). In addition to LTRs, DNA transposons including CMC-EnSpm (1.8%), PIF (2.6%), MULE (7.4%), hAT (7.8%), and helitron (15.3%) were also abundant. This result suggests that TEs have transcriptional activity and proliferate in *P. ginseng* tissues, thus playing an important role in genome and gene evolution by controlling transposition at a post-transcriptional level [37]. We also identified a total of 60,304 simple sequence repeats (SSRs) in unigenes (Figure 3C), more than the 13,044 SSRs previously reported [38]. The SSR motifs in di- and tri-nucleotide repeats were primarily AG (accounting for 53.9% of dinucleotide repeats) and AAG (24.3% of trinucleotide repeats) sequences. Di- and tri-nucleotide repeats were also predominantly identified in untranslated regions (UTRs) and ORFs. This result indicates that the *P. ginseng* transcribed regions are characterized by SSRs.

### 3.3. Efficient Gene Annotation of P. ginseng

**Figure 4:**
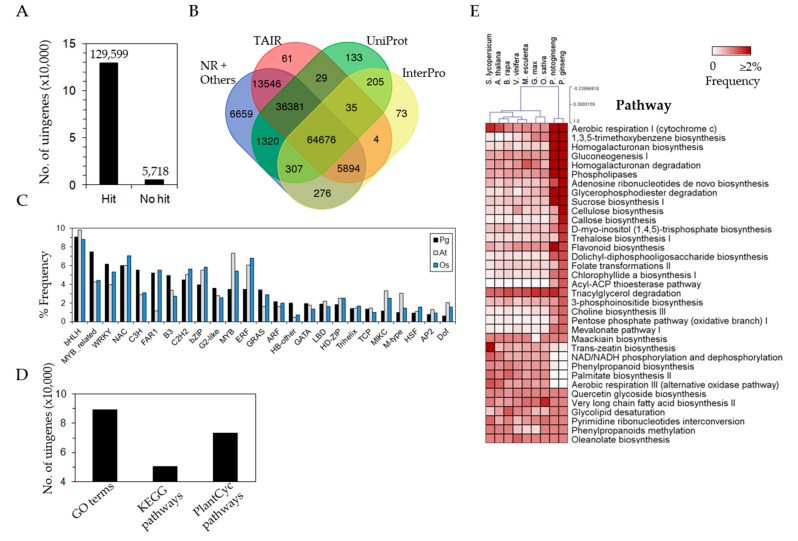
Functional annotation of unigenes. (**A**) The number of unigenes hits to known sequences. (**B**) Overlap of hits among known homologous genes searched against the different databases including UniProt, NR, TAIR, and InterPro. (**C**) The primary transcription factor (TF) families identified in unigenes. (**D**) The number of unigenes assigned to functional categories. (**E**) Abundance of unigenes assigned to the primary Plant Metabolic Pathways (PMN). Abundances of genes in *Arabidopsis thaliana*, *Manihot esculenta*, *Brassica rapa*, *Vitis vinifera*, *Oryza sativa*, *Glycine max*, *Solanum lycopersicum*, *P. ginseng* and *P. notoginseng* assigned to the PMN pathway were compared and represented in a heatmap. Pg: *P. ginseng*; At: *A. thaliana*; Os: *Oryza sativa*.

Obtaining complete or longer ORFs using Iso-Seq increases the efficiency of functional gene prediction or annotation, especially in the absence of reference genome information. To date, de novo transcriptome assembly using Illumina RNA-Seq data has been limited to more accurate and longer contig assembly because of short read lengths, resulting in chimeric contigs and/or low gene annotation. Of all unigenes called by Iso-Seq, 129,599 unigenes (95.8%) were aligned to protein sequence databases such as UniProt, NR, TAIR, and InterPro using BLAST and InterProScan (Figure 4A), demonstrating outstanding hit scores within the protein sequence databases. In particular, 64,676 unigenes (47.8%) were predicted as known homologous genes by searching within databases (Figure 4B), and 120,626 of these annotated unigenes showed homology to 14,403 genes in *Arabidopsis* (Figure 4B). A total of 52,920 transcription factor (TF)-related sequences were also matched against PlantTFDB using BLAST, corresponding to 1488 TF-related Pfam domains. MYB-related TFs, WRKY, C3H, B3, and HB families in *P. ginseng* were abundant compared with the *Arabidopsis* and rice genomes, although the frequencies of TF families varied (Figure 4C). The annotated unigenes were assigned to GO categories (89,239 unigenes), KEGG (50,239 unigenes), and Plant Metabolic Pathways (73,453 unigenes) (Figure 4D). The Plant Metabolic Pathways revealed the functional categories of highly abundant genes in *P. ginseng* compared to seven model plant genomes (Figure 4E) and included: aerobic respiration, 1,3,5-trimethoxybenzene biosynthesis, homogalacturonan biosynthesis, gluconeogenesis, homogalacturonan degradation, phospholipases, adenosine ribonucleotides de novo biosynthesis, glycerophosphodiester degradation, sucrose biosynthesis, cellulose biosynthesis, callose biosynthesis, d-myo-inositol (1,4,5)-trisphosphate biosynthesis, trehalose biosynthesis, flavonoid biosynthesis, dolichyl-diphosphooligosaccharide biosynthesis, folate transformations, chlorophyllide a biosynthesis, acyl-ACP thioesterase pathway, triacylglycerol degradation, 3-phosphoinositide biosynthesis, choline biosynthesis, pentose phosphate pathway, mevalonate pathway, and maackiain biosynthesis. Interestingly, the abundance of protein-coding genes involved in these pathways of *P. ginseng* was highly similar to those of *P. notoginseng,* even though slight variation was observed (Figure 4E). However, flavonoid biosynthesis and triacylglycerol degradation were more abundant in *P. notoginseng* (Figure 4E).

To date, candidate genes involved in triterpenoid saponin synthesis have been identified and are critical enzyme-encoding genes from *P. ginseng* [9,39]. However, low coverage and/or partial short contig assembly in transcriptome sequencing hinders the identification of such candidates. In this study, we identified 314 unigenes previously reported to be involved in triterpenoid saponin synthesis [9,39] (Table 1). Remarkably, of those genes, 233 unigenes (74.2%) were predicted to contain complete ORFs. β-amyrin synthase and β-amyrin 28-oxidase, key upstream molecules involved in triterpenoid saponin synthesis, were expressed as five and two paralogs in complete ORFs. Moreover, squalene synthase, squalene epoxidase, and dammarenediol-II synthase appear as families containing several paralogs with alternative splicing isoforms. We also analyzed the number of homologous genes involved in triterpene saponin biosynthesis between *P. notoginseng* and *P. ginseng* by using TBLASTX with a cutoff ≤10^−10^ (Table S4). Compared to the candidate genes of *P. notoginseng*, those of *P. ginseng* appear to be overestimated despite of showing high similarities. These results would be come from abundance of sequence variation in our assembled unigenes which can generate different isoform clusters and increase the number of unigenes. It is also possible that sequence heterozygosity by pooling four different tissues may increase different isoform clusters. These results suggest that our data will be an invaluable resource for identifying candidate genes and understanding functional pathways involved in triterpenoid saponin synthesis.

### 3.4. Identification and Functional Analysis of Auxin and Cytokinin Signaling Pathways in P. ginseng

**Figure 5:**
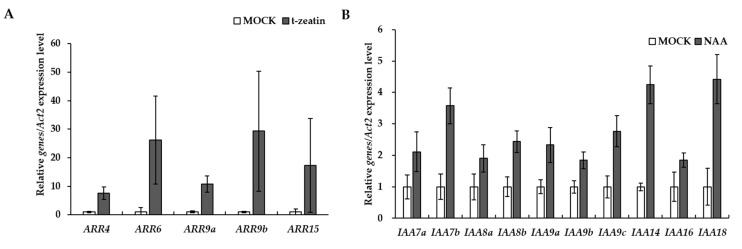
Negative feedback loops in auxin and cytokinin signaling responses in *P. ginseng* seedlings. Relative gene expression levels of type-A response regulators (**A**) and AUX/IAAs (**B**) in three-month-old *P. ginseng* seedlings treated with cytokinin and auxin, respectively, were quantified by a real time qRT-PCR (*n* = 4, Error bars indicate ± standard error).

Plant hormones are key players in plant growth and developmental processes. Among the nine phytohormones studied, auxin and cytokinin have fundamental roles in embryogenesis and meristem development. Recent studies have revealed essential functions of auxin and cytokinin’s synergistic or antagonistic interactions in the regulation of plant growth and other developmental processes [40,41]. However, neither annotation nor functional studies of genes related to auxin or cytokinin signaling or biosynthetic pathways in ginseng plants are available. As shown in Table 2, we identified a total of 354 complete ORFs of putative auxin signaling-related genes, including SCF^TIR/AFBs^ receptor complexes (47 unigenes), Auxin Response Factors as key transcription factors (274 unigenes) and AUX/IAAs negative regulators (33 unigenes). We also screened 53 complete ORFs that encoded cytokinin-related two-component signaling genes (Table 3). To test whether the common canonical signaling pathways of auxin and cytokinin are also evolutionary conserved in *P. ginseng*, we surveyed the negative feedback loop effects in these signaling responses. As previously reported, rapid upregulation of AUX/IAAs and type-A response regulators, which were identified as negative feedback loops in auxin and cytokinin signaling, respectively, was observed in the presence of the corresponding phytohormones (Figure 5). These results suggest that the transcriptome database is useful for the identification of candidate genes and to understand their functionality in *P. ginseng*.

### 3.5. Identification of Alternative Splicing Isoforms

**Figure 6:**
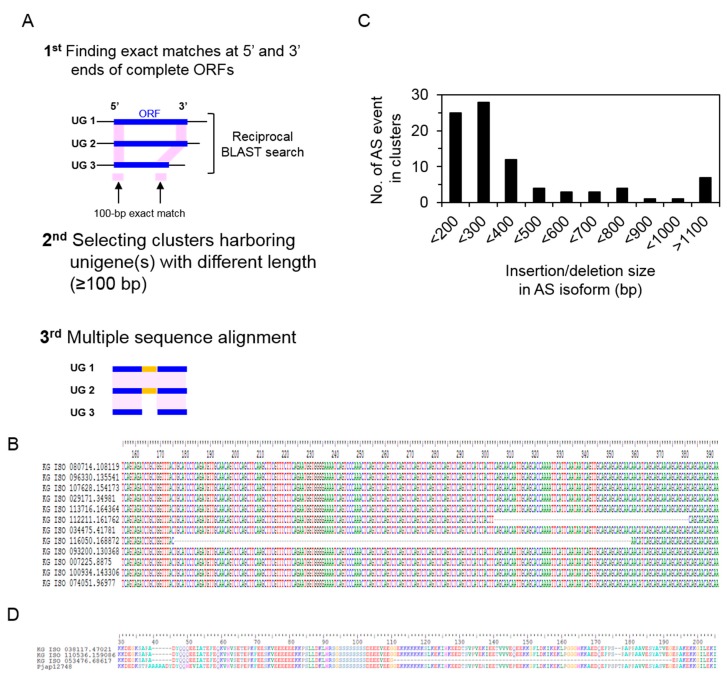
Identifications of alternative splicing isoforms within unigenes (UG) in *P. ginseng*. (**A**) Workflow for identifying alternative splicing (AS) events within unigenes. (**B**) Identifications of AS isoforms of *ARF6* homologues. A total of 12 *ARF6* homologous unigenes were clustered, and two regions showing of 78 (KG ISO 112211) and 183 (KG ISO 116050) base pair deletions were found. (**C**) The size of insertions/deletions (InDels) by AS events and the number of corresponding AS events in clusters. (**D**) Conservation of *ERD14* between *P. ginseng* and *P. notoginseng*, and identifications of AS isoforms in *P. ginseng*.

We also confirmed that a total of 88 clusters contained alternative splicing events (Figure 6). Because whole-genome de novo assembly data of *P. ginseng* are not yet available, we analyzed alternative splicing events for internal exon(s), such as exon skipping, within unigenes (but not in the 5- or 3′-ends of unigenes). Unigenes that were exact matches for both 100 bases at the 5′ and 3′ ends within their complete ORFs were clustered, and those exhibiting insertions/deletions of more than 100 bases were selected and validated by multiple-sequence alignments (Figure 6A). For example, an exon skipping event with a 183-base deletion was detected in the *ARF6* homolog (auxin response factor 6, Figure 6B). In addition, a 78-base deletion was found in the unigene cluster that was translated even though it was not identified as having alternative splicing isoforms. Insertions/deletions (InDels) were mostly distributed in the size range of 100 bp to 400 bp (Figure 6C), and resulted in the variation of protein translation. These clusters might result from tissue-specific expression, explaining the differences in amino acid sequence and in biological function. Those clusters were also compared with homologous genes in *P. notoginseng*. A total of seven clusters were identified by multiple sequence alignment with high sequence identity, and InDels were found only in *P. ginseng* at the level of protein sequences, suggesting the modification of gene structures in *P. ginseng* (Figure 6D). Future studies that use diverse strategies to assess genome-wide transcriptomics in *P. ginseng* will provide insight into the growth and developmental features of this important plant. 

## 4. Discussion

Transcriptome data generated by Iso-Seq generate longer and improved unigenes from *P. ginseng* with a high level of assembly completeness and gene annotation, enabling a comprehensive view of the transcriptome. Conventional methods, such as cDNA cloning and expressed sequence tags (EST) sequencing, have limitations and do not efficiently provide accurate sequence information, including expressed mRNA. Although high-throughput sequencing using the Illumina HiSeq platform has recently produced genome-wide transcriptome data with good sequencing depth and coverage [13], de novo transcriptome assembly using short reads have generated short and partial transcript contigs containing artifacts including chimeras, structural errors, incomplete assembly, and base errors, resulting in a high misassembly rate and unreliable gene annotation [42]. In these cases, it has been almost impossible to elucidate the function of genes associated with traits of interest as well as novel transcripts. To avoid such limitations in *P. ginseng*, we first collected a large portion of full-length ORF containing transcriptome data from four independent tissues (root, stem, leaf, and flower), which maximizes transcript diversity, and used the PacBio SMRT sequencing approach. As expected, a large amount of transcriptome data was generated, including 135,317 unigenes with 91.94% high assembly completeness that were much larger (0.79 to 1.9 kb) than reported previously [13,36,39]. Moreover, a reference for quantifying gene expression in the absence of reference genome information is likely to be very useful with high mapping rate (Figure 2C and Figure S9). For this, genome-wide coverage of transcripts should be strengthened. Our data were not relatively well-covered for fruit tissue-related expressed genes (Figure 2C) due to absence of RNA extraction from reproductive ginseng fruits. Moreover, considerable small size transcripts less than approximately 1 kb seem to be missed (Figure S4). This problem appears to result from technical limitations of a PacBio sequencing platform associated with size selection in the construction of mRNA sequencing libraries. From the observations, Illumina RNA-Seq data are likely to be effective for the coverage of expressed genes, especially small size transcripts, in the genome, and PacBio Iso-Seq data are useful for the verification of gene prediction with full-length open reading frames (FL-ORF), thus suggesting combinatorial data analysis in de novo transcriptome assembly. In spite of such defect, Iso-Seq data increases the efficiency of functional gene prediction or annotation by the finding of FL-ORFs. In the result, 64,676 (47.8%) unigenes out of 135,317 isoforms identified were successfully annotated as known homologous genes using BLAST searches of the NR, TAIR, Swiss-Prot and InterProScan databases (Figure 4). This implies that more than half of unigenes generated in this study were not annotated according to existing databases. This could be as a result of several reasons such as the absence of reference genomic information on the family Araliaceae and the unigenes without hits probably belonged to untranslated regions. It is also possible that they could contain non-coding RNA and short sequences absenting protein functional domains. Since lacking genomic and transcriptomic information in the family Araliaceae, these unigenes without hits may be considered putative novel transcribed sequences. Therefore, according to these results, there is a need to generate a large collection of unigenes and further characterize the gene structures and expression patterns in *P. ginseng*. Furthermore, as previously reported [21], the PacBio isoform data could provide much longer transcript, isoform and gene structure information than the preexisted genome databases

Full-length transcript sequence information with complete ORF structures is valuable for gene annotation and functional genomics in plants. FL information provides a more effective approach for discovering candidate genes involved in secondary metabolite biosynthesis. In our study, we identified 315 unigenes related to triterpenoid saponin biosynthesis and 233 unigenes predicted to harbor complete ORFs. Although previous studies also identified candidates related to triterpenoid saponin biosynthesis [13,43], most of the sequences described were partial transcripts from root tissues. Based on SMRT sequencing and analysis of the four different types of *P. ginseng* tissues, we have found the 16 genes with complete ORFs encoding key enzymes involved in ginsenoside backbone biosynthesis, including squalene epoxidase, dammarenediol-II synthase and farnesyl diphosphate synthase (Table 1). Therefore, these results strongly indicate that most of the genes involved in the synthesis of ginsenosides are contained within our transcriptome data. Accordingly, our results will provide a foundation for understanding the molecular mechanism of ginsenoside biosynthesis in *P. ginseng* plants.

As represented in Figure 4, a comparative analysis of transcription factor gene structure showed that when compared to *Arabidopsis* and rice, *P. ginseng* contained a higher proportion of transcription factors that control secondary metabolism, cell morphogenesis, and transmission of hormone and stress signals, including MYB, WRKY, C3H, B3, and HB. MYB and WRKY are involved within the synthesis of the secondary metabolites of ginseng [44,45]. C3H, B3, and HB are responsible for precisely controlling gene expression in *Arabidopsis* and rice following environmental changes [46,47]. Understanding the metabolic pathways for ginseng secondary metabolite synthesis and identifying the transcription factors controlling related genes have economic importance because the activity and quality of plant secondary metabolite synthetic pathways are directly linked. Overall, our transcriptome data generated using long-read sequencing exhibited the following features: (1) identification of much more portion of complete ORF structures; (2) a high level of gene annotation with efficient identification of genes associated with useful traits such as triterpenoid saponin and auxin/cytokinin signaling; (3) abundance of TEs with transcriptional activity in *P. ginseng*; and (4) identification of alternative splicing isoforms encoded by single genes. Moreover, when other RNA-Seq data were compared with our data, a high mapping rate was revealed (Figure 2), which will be a valuable transcriptomics resource. Therefore, our results will contribute to studies on gene function and will hasten the completion of a reference genome for *P. ginseng*.

Some unigenes in our dataset were exactly matched by comparing with the Sanger sequencing (Figure 2B). These results indicated that the SMRT sequencing approach is reliable for generating high-quality, full-length sequence data from *P. ginseng*. Accordingly, we expect that these long-read transcripts will provide the accuracy of transcriptome characterization compared with transcript tags assembled from short RNA-seq reads. Additionally, our survey of plant hormonal signaling-related genes (Table 2 and Table 3) and their expression patterns from upstream signal cues suggested that our annotations can predict the function of specific *P. ginseng* genes (Figure 5 and Figure S8). Recent advances in molecular genetic studies for crop plants have revealed that key developmental and hormone-related genes are closely linked to diverse quantitative traits and play critical roles in domestication during breeding and artificial selection [48]. Interestingly, large number of transcription regulators of auxin and cytokinin signaling pathways is presented in *P. ginseng* transcriptome (Table 2 and Table 3). Complex gene networks would be able to increase the cope of efficiency to diverse environmental changes during long terms of growth and developmental processes of this plant. Therefore, the functional identification of key genes and their signaling networks in *P. ginseng* will be helpful for developing biotechnological approaches to enhance the quality and consumer-friendly market of *P. ginseng* and related medicinal plants. Taken together, our Iso-Seq data will serve as a valuable resource for understanding the metabolic pathways related to triterpenoid saponin synthesis as well as growth and developmental signaling pathways. In addition, our results will improve the development of ginseng varieties with increased ginsenoside content and enhanced resistance to environmental stress.

## Figures and Tables

**Table 1:** Unigenes involved in triterpene saponin biosynthesis in *P. ginseng*.

Gene Name & Description	EC Number	Number of Unigene	Number of Unigene with Complete ORF
*AACT*, acetyl-CoA acetyltransferase	2.3.1.9	18	12
*HMGS*, hydroxymethylglutaryl-CoA synthase	2.3.3.10	22	18
*HMGR*, hydroxymethylglutaryl-CoA reductase	1.1.1.34	35	26
*MVK*, mevalonate kinase	2.7.1.36	13	10
*PMK*, phosphomevalonate kinase	2.7.4.2	17	10
*MVD*, mevalonate diphosphate decarboxylase	4.1.1.33	8	5
*GGPPS*, geranylgeranyl pyrophosphate synthase	2.5.1.29	17	14
*FPPS*, farnesyl diphosphate synthase	2.5.1.10	36	23
*IPPI*, isopentenyl diphospate isomerase	5.3.3.2	7	2
*SS*, squalene synthase	2.5.1.21	15	14
*SE*, squalene epoxidase	1.14.99.7	64	47
*DS*, dammarenediol-II synthase	4.2.1.125	33	29
*β-AS*, β-amyrin synthase	5.4.99.39	5	5
*β-A28O*, β-amyrin 28-oxidase	1.14.13	2	2
*D12H*, dammarenediol 12-hydroxylase	1.14.13.183	13	9
*P6H*, protopanaxadiol 6-hydroxylase	1.14.13.184	9	7

**Table 2:** Unigenes involved in auxin signal components in *P. ginseng*.

Gene Name	[TAIR] Description	[TAIR] AGI Number	Number of Unigenes	Number of Unigene with Complete ORF
Aux/IAA				
*IAA7*	Indole-3-acetic acid 7	AT3G23050.1	3	2
*IAA8*	Indoleacetic acid-induced protein 8	AT2G22670.4	18	10
*IAA9*	Indole-3-actic acid inducible 9	AT5G65670.1	21	17
*IAA14*	Indole-3-acetic acid inducible 14	AT4G14550.1	1	1
*IAA16*	Indoleacetic acid-induced protein 16	AT3G04730.1	2	1
*IAA17*	Indole-3-acetic acid inducible 17	AT1G04250.1	2	1
*IAA18*	Indole-3-acetic acid inducible 18	AT1G51950.1	2	1
SCF complex				
*SKP1*	S phase kinase-associated protein 1	AT1G75950.1	11	7
*CUL1*	Cullin 1	AT4G02570.1	55	38
*AFB1*	Auxin signaling F box protein 1	AT4G03190.1	5	2
Auxin Response Factor				
*ARF3*	Auxin response factor 3	AT2G33860.1	17	15
*ARF5*	Auxin response factor 5	AT1G19850.1	16	10
*ARF6*	Auxin response factor 6	AT1G30330.2	146	127
*ARF7*	Auxin response factor 7	AT5G20730.1	19	9
*ARF8*	Auxin response factor 8	AT5G37020.1	41	38
*ARF16*	Auxin response factor 16	AT4G30080.1	30	27
*ARF17*	Auxin response factor 17	AT1G77850.1	4	3
*ARF19*	Auxin response factor 19	AT1G19220.1	56	45

**Table 3:** Unigenes involved in cytokinin signal components in *P. ginseng*.

Gene Name	[TAIR] Description	[TAIR] AGI Number	Number of Unigenes	Number of Unigene with Complete ORF
**Histidine Kinase**				
*AHK2*	Arabidopsis histidine kinase 2	AT5G35750.1	3	2
*AHK3*	Arabidopsis histidine kinase 3	AT1G27320.1	5	5
*AHK4*	Arabidopsis histidine kinase 4	AT2G01830.2	5	5
**Histidine-Containing Phosphotransmitter**				
*AHP1*	HP 1	AT3G21510.1	2	2
*AHP5*	HP 5	AT1G03430.1	1	1
**Type A-Response Regulator**				
*ARR4*	Response regulator 4	AT1G10470.1	1	1
*ARR6*	Response regulator 6	AT5G62920.1	1	0
*ARR9*	Response regulator 9	AT3G57040.1	2	2
*ARR15*	Response regulator 15	AT1G74890.1	1	0
**Type B-Response Regulator**				
*ARR1*	Response regulator 1	AT3G16857.2	4	4
*ARR2*	Response regulator 2	AT4G16110.1	19	19
*ARR10*	Response regulator 10	AT4G31920.1	2	2
*ARR11*	Response regulator 11	AT1G67710.1	3	3
*ARR12*	Response regulator 12	AT2G25180.1	5	5
*ARR18*	Response regulator 18	AT5G58080.1	2	2

## Supplementary Materials

The following are available online at www.mdpi.com/2073-4425/8/9/228/s1. Figure S1: The distribution of read length of consensus isoforms. Figure S2: Summary of transcript assemblies along with sequence coverage and identity for clustering of all the libraries. Figure S3: The length distribution of unigenes and predicted ORFs. Figure S4: Mapping of unigenes (query) from Illumina RNA-Seq reported by Wang et al. (2016) [13] to unigenes (subject) from Iso-Seq. Figure S5: The length distribution of unigenes generated by Illumina RNA-Seq (green) and PacBio Iso-Seq (blue). Figure S6: Mapping of Illumina RNA-seq dataset 1 to the two unigene sets from Iso-Seq and Illumina RNA-Seq dataset 1 using STAR. Figure S7: Abundance of TEs in *P. gineng* unigenes. Figure S8: Identification of plant hormone signal components. Figure S9: Mapping of Illumina RNA-Seq dataset 1 reported by Wang et al. (2016) [13] to Iso-Seq unigene dataset as a reference. Table S1: Summary of subreads after removal of adapters and artifacts. Table S2: Classification of isoform clusters. Table S3: Summary of consensus isoforms. Table S4: List of protein-coding genes of *P. notoginseng* homologous to unigenes involved in triterpene saponin biosynthesis in *P. ginseng*. Table S5: Identification of alternative splicing (AS) events in unigenes of *P. ginseng*. Table S6: Primer lists for qRT-PCR.
