# Supplementary material for: Isoform Sequencing Provides a More Comprehensive View of the Panax ginseng Transcriptome
Source: Genes (Basel). 2017 Sep 15;8(9):228. doi: 10.3390/genes8090228 (PMC5615361; doi:10.3390/genes8090228)
Supplement: Supplementary file 1 [file genes-08-00228-s001.pdf]

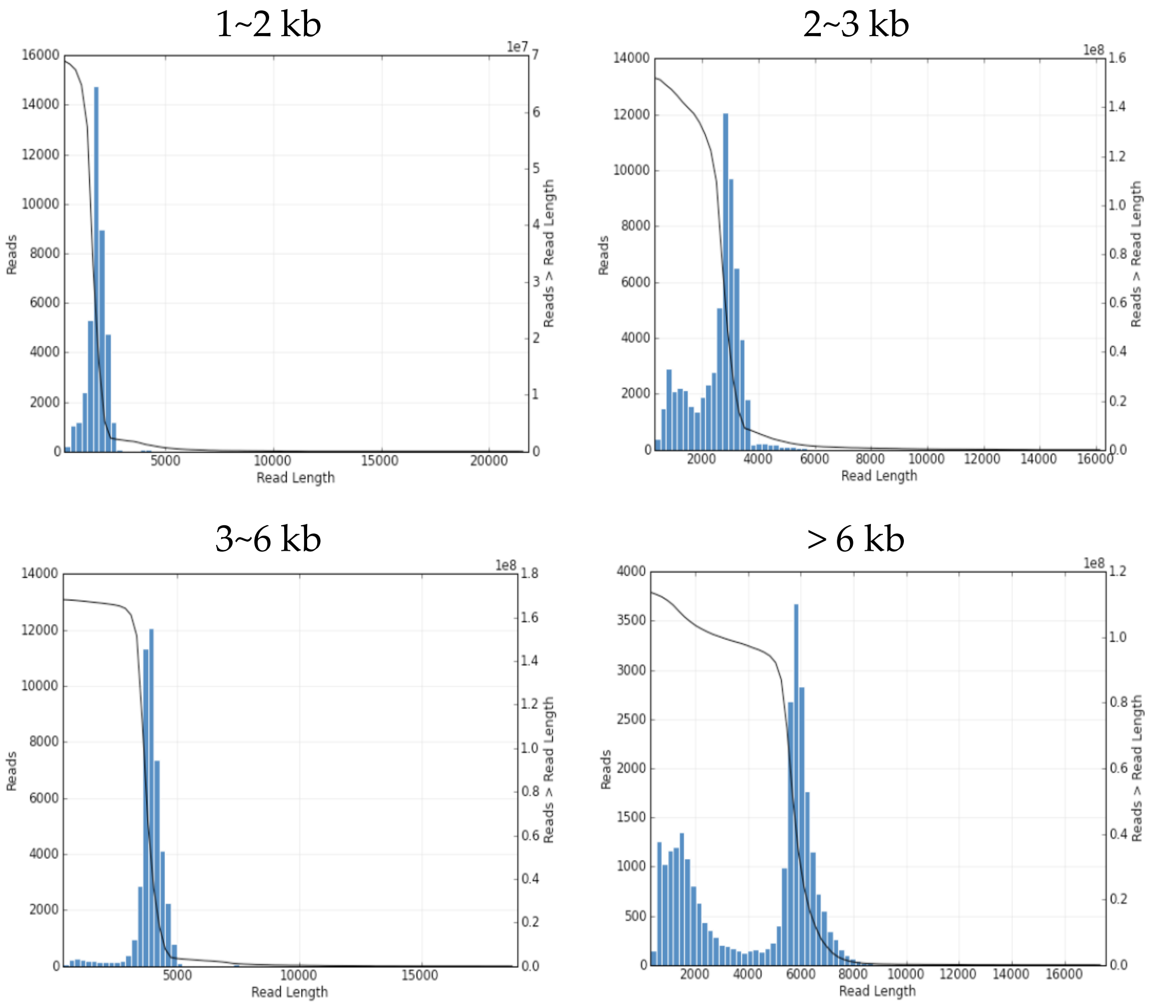


**Figure S1. The distribution of read length of consensus isoforms.**

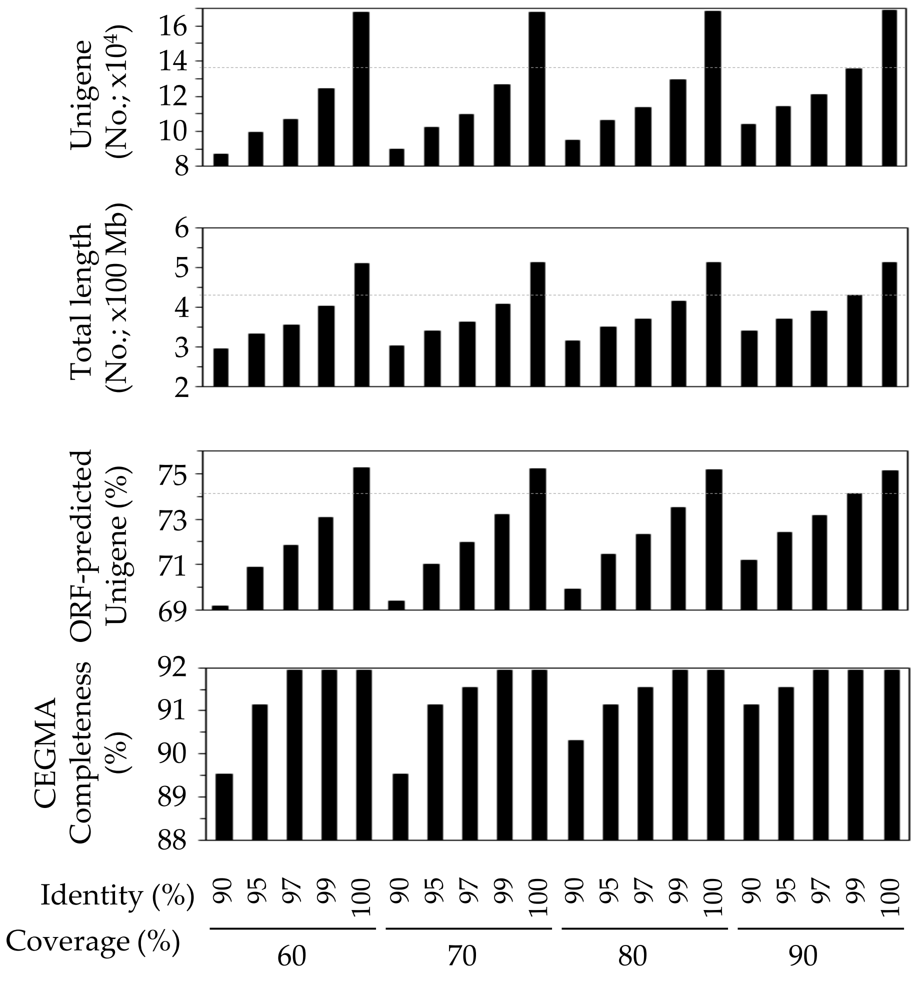


**Figure S2. Summary of transcript assemblies along with sequence coverage and identity for clustering of all the libraries.** The summary includes the number of unigenes (No.), total length (bases), the number of unigenes harboring ORFs predicted (No.), and CEGMA completeness (%)

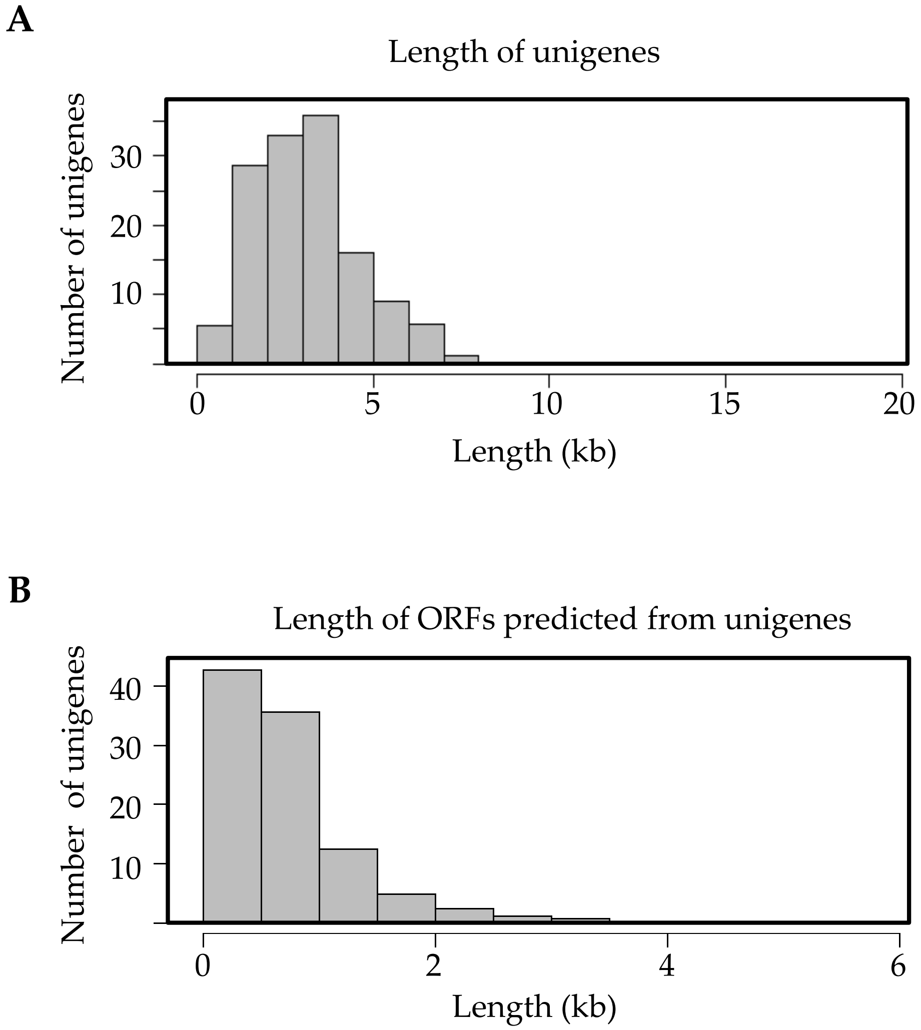


**Figure S3. The length distribution of unigenes (A) and predicted ORFs (B).**

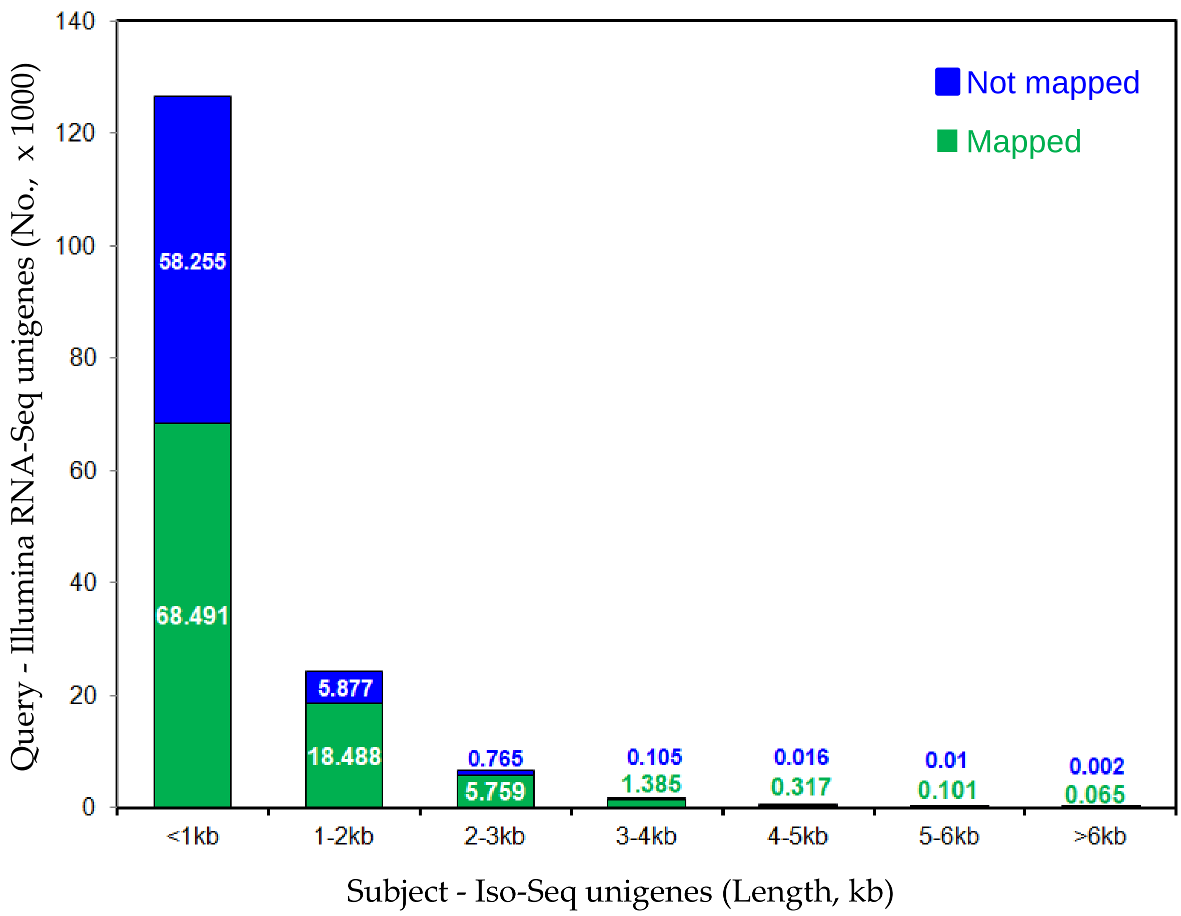


**Figure S4. Mapping of unigenes (query) from Illumina RNA-Seq reported by Wang et al. (2016) to unigenes (subject) from Iso-Seq.**

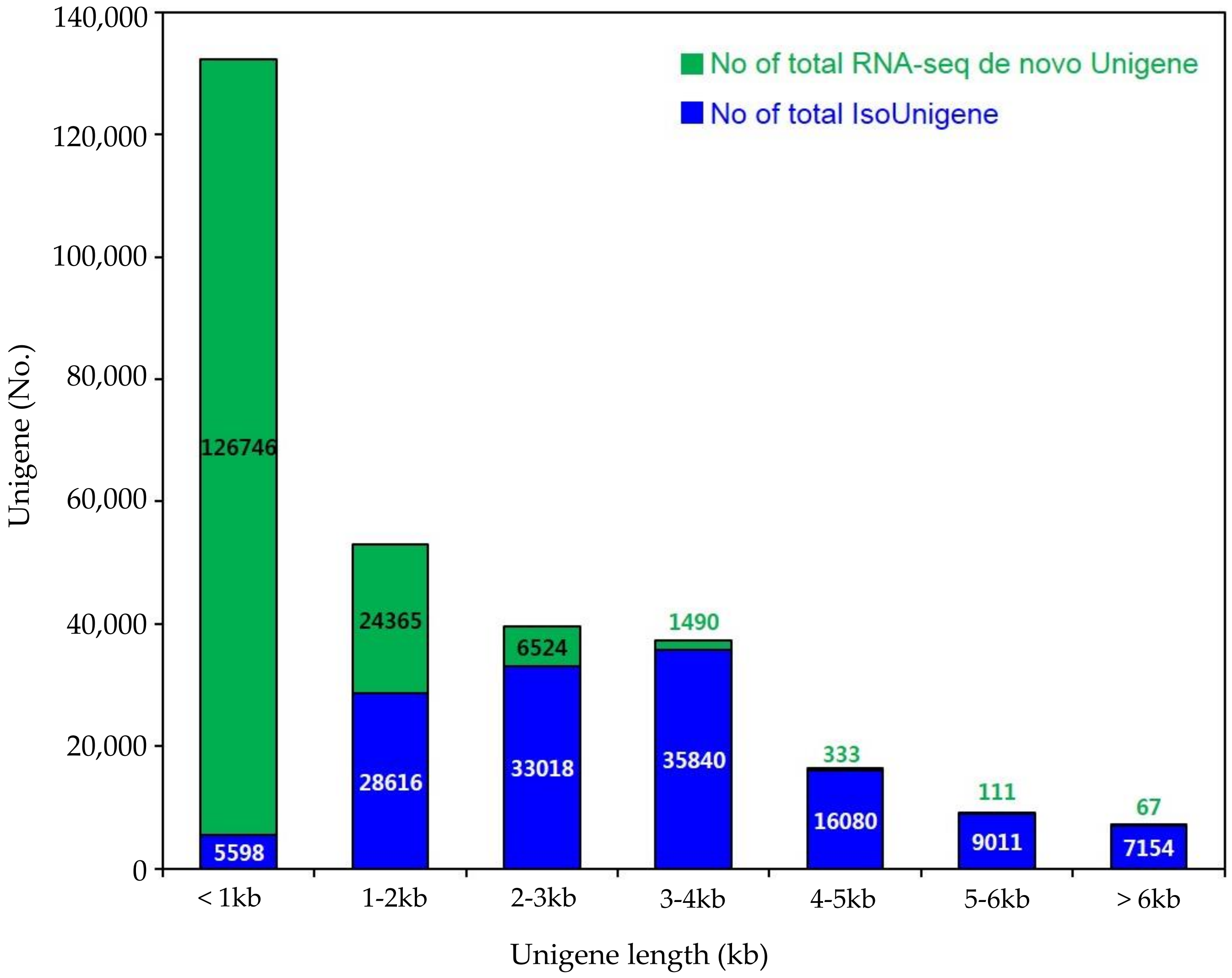


**Figure S5. The length distribution of unigenes generated by Illumina RNA-Seq (green) and PacBio Iso-Seq (blue).**

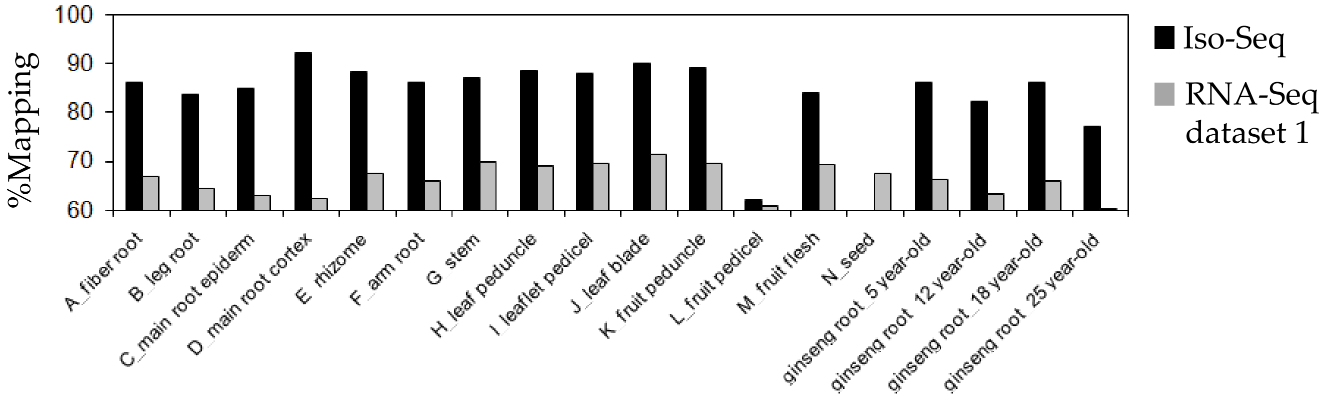


**Figure S6. Mapping of Illumina RNA-seq dataset 1 to the two unigene sets from Iso-Seq and Illumina RNA-Seq dataset 1 using STAR.**

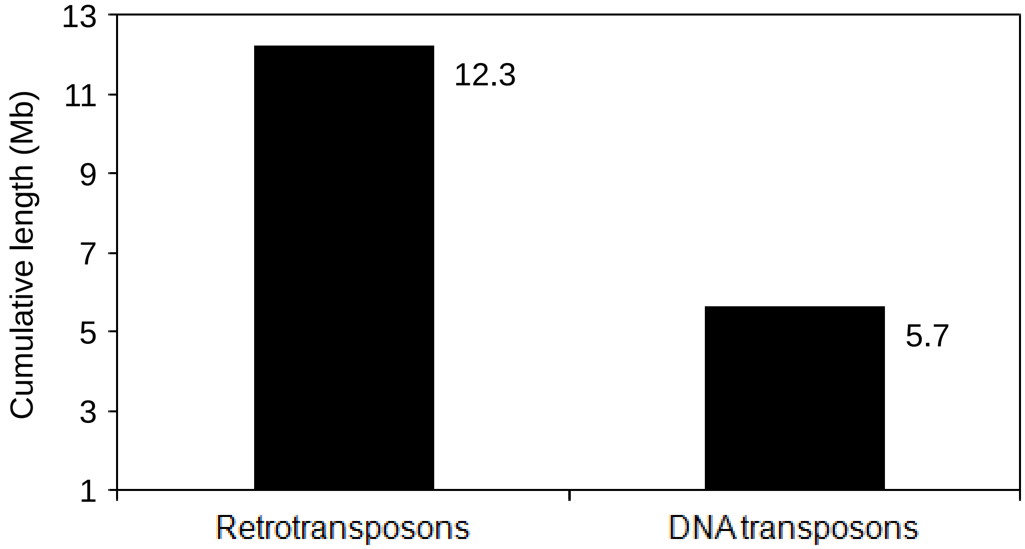


**Figure S7. Abundance of TEs in *P. gineng* unigenes**

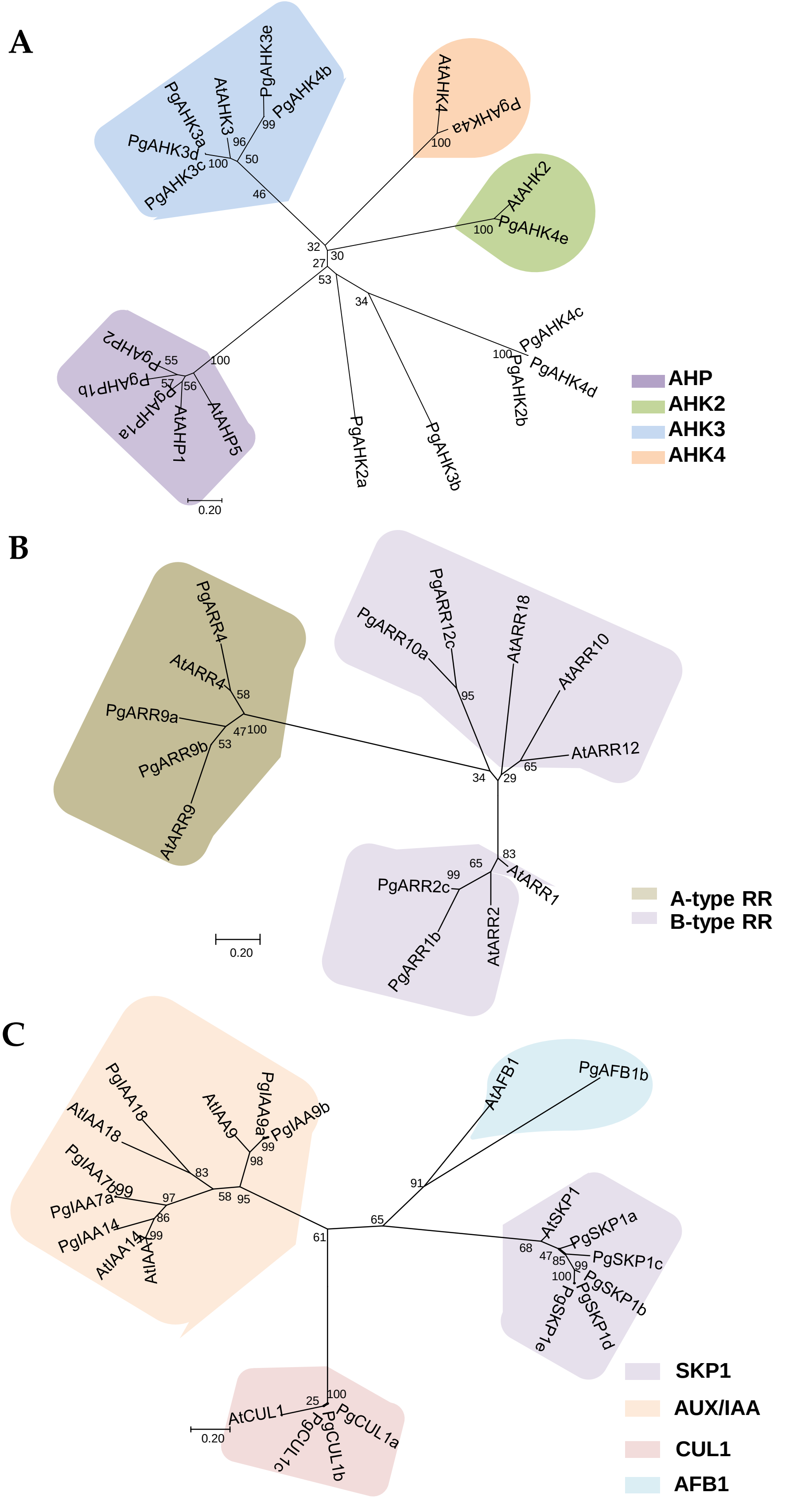


**Figure S8.** Identification of plant hormone signal components including cytokinin related two-component genes (histidine kinases and histidine phosphor transmitter (A) and respose regulators (B)) and auxing signaling components (C) in the *P. ginseng* transcriptome.

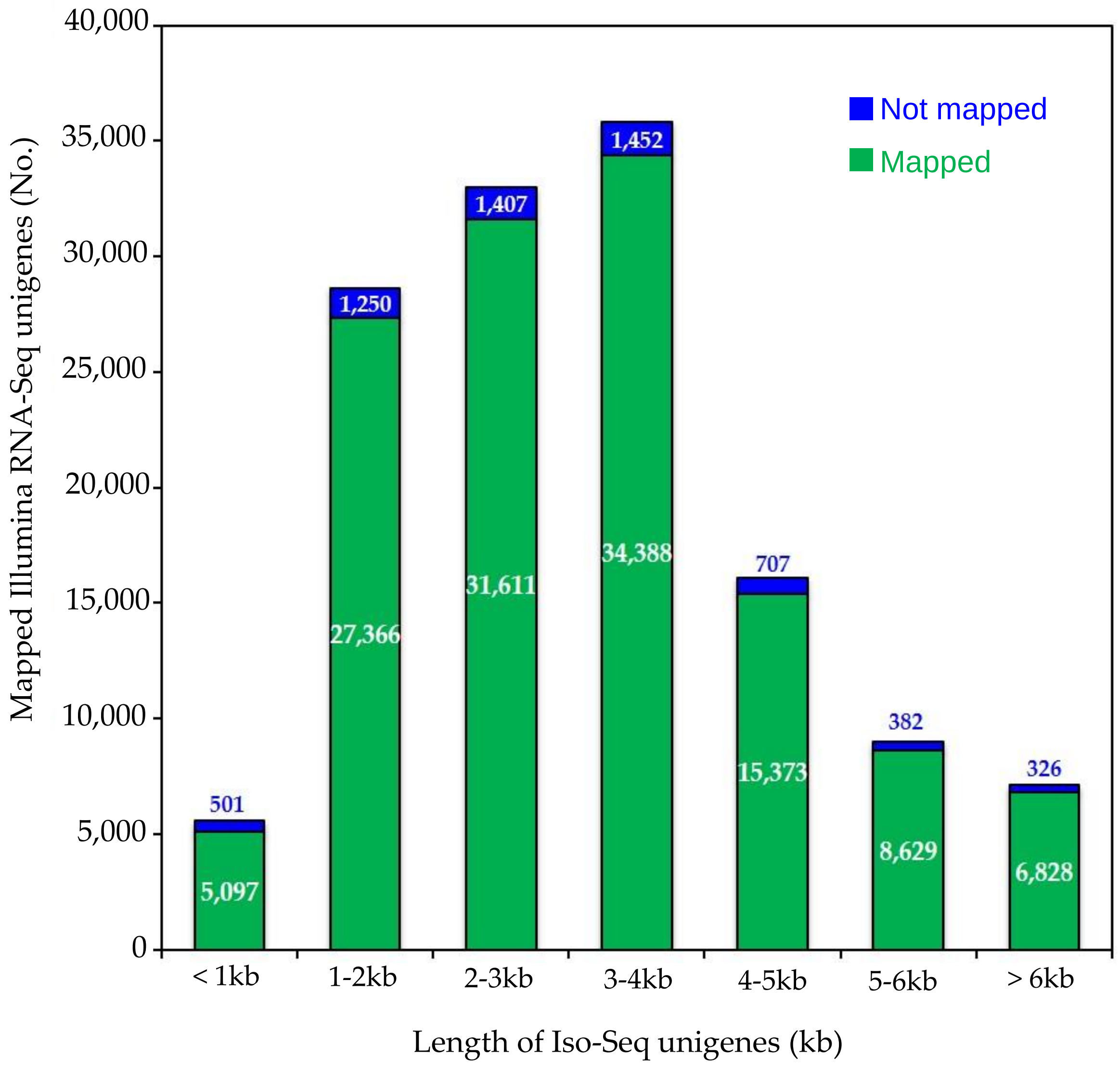


**Figure S9. Mapping of Illumina RNA-Seq dataset 1 reported by Wang et al. (2016) to Iso-Seq unigene dataset as a reference.**

**Table S1.** Summary of subreads after removal of adapters and artifacts

| Library size | Replicate | Number of subreads | Total length of subreads (Gb) |
|---|---|---|---|
| 1~2 kb | 1 | 635,397 | 1.30 |
| | 2 | 555,922 | 1.12 |
| | 3 | 555,496 | 1.13 |
| | 4 | 583,622 | 1.22 |
| 2~3 kb | 1 | 772,914 | 1.75 |
| | 2 | 703,751 | 1.63 |
| | 3 | 677,792 | 1.57 |
| | 4 | 720,334 | 1.66 |
| 3~6 kb | 1 | 414,630 | 1.59 |
| | 2 | 442,486 | 1.64 |
| | 3 | 410,099 | 1.54 |
| | 4 | 420,446 | 1.62 |
| >6 kb | 1 | 311,013 | 1.43 |
| | 2 | 356,066 | 1.53 |
| | 3 | 319,438 | 1.37 |
| | 4 | 347,931 | 1.42 |
| Total | | 8,227,337 | 23.52 |

**Table S2.** Classification of isoform clusters

| | 1~2 kb | 2~3 kb | 3~6 kb | >6 kb |
|---|---|---|---|---|
| Number of reads of insert | 182,241 | 247,189 | 192,206 | 163,195 |
| Number of 5`-reads | 110,658 | 160,459 | 114,426 | 62,466 |
| Number of 3`-reads | 107,067 | 163,149 | 118,265 | 64,507 |
| Number of poly-A reads | 98,681 | 152,078 | 116,280 | 58,703 |
| Number of filtered short reads | 10,832 | 8,159 | 2,365 | 2,882 |
| Number of non-full length reads | 82,801 | 107,312 | 95,446 | 113,549 |
| Number of full-length reads | 88,608 | 131,718 | 97,395 | 446,764 |
| Number of full-length non-chimeric reads | 87,935 | 131,191 | 96,491 | 45,384 |
| Average full-length non-chimeric read length | 1,845 | 2,644 | 3,865 | 4,723 |

**Table S3.** Summary of consensus isoforms

| | 1~2 kb | 2~3 kb | 3~6 kb | >6 kb |
|---|---|---|---|---|
| Number of consensus isoforms | 40,390 | 62,217 | 44,783 | 27,327 |
| Average read length of consensus isoforms | 1,843 | 2,543 | 3,871 | 4,271 |
| Number of polished high-quality isoforms | 28,365 | 43,281 | 20,178 | 11,275 |
| Number of polished low-quality isoforms | 11,914 | 18,426 | 24,603 | 15,868 |
| Total number of polished isoforms | 40,279 | 61,707 | 44,781 | 27,143 |

**Table S4.** List of protein-coding genes of *P. notoginseng* homologous to unigenes involved in triterpene saponin biosynthesis in *P. ginseng*

| Gene name | No. of unigene of *P. ginseng* | No. of hit gene of *P. notoginseng* | Genes of *P. notoginseng* (ID) | Best hit *E*-value (BLASTX) |
|---|---|---|---|---|
| AACT, Acetyl-CoA acetyltransferase | 18 | 2 | Pjap02127<br>Pjap28751 | 0<br>1E-166 |
| HMGS, Hydroxymethylglutaryl-CoA synthase | 22 | 3 | Pjap05414<br>Pjap33244<br>Pjap31618 | 0<br>1E-154<br>7E-121 |
| HMGR, Hydroxymethylglutaryl-CoA reductase | 35 | 5 | Pjap29075<br>Pjap20004<br>Pjap25684<br>Pjap21692<br>Pjap10209 | 0<br>5E-145<br>3E-60<br>4E-152<br>2E-30 |
| MVK, Mevalonate kinase | 13 | 1 | Pjap02763 | 0 |
| PMK, Phosphomevalonate kinase | 17 | 5 | Pjap26215<br>Pjap05842<br>Pjap00173<br>Pjap19214<br>Pjap05278 | 7E-72<br>7E-41<br>8E-36<br>9E-20<br>1E-17 |
| MVD, Mevalonate diphosphate decarboxylase | 8 | 2 | Pjap30796<br>Pjap01306 | 3E-117<br>2E-17 |
| GGPPS, Geranylgeranyl pyrophosphate synthase | 17 | 5 | Pjap18977<br>Pjap18534<br>Pjap08347<br>Pjap04663<br>Pjap30381 | 4E-19<br>0<br>0<br>3E-157<br>9E-169 |

| | | | | |
|---|---|---|---|---|
| FPPS,<br>Farnesyl diphosphate synthase | 36 | 1 | Pjap26546 | 0 |
| IPPI,<br>Isopentenyl diphospate isomerase | 7 | 5 | Pjap02090<br>Pjap12765<br>Pjap03732<br>Pjap31081<br>Pjap07103 | 1E-83<br>7E-36<br>2E-117<br>3E-25<br>2E-10 |
| SS,<br>Squalene synthase | 15 | 2 | Pjap06586<br>Pjap25515 | 0<br>7E-111 |
| SE,<br>Squalene epoxidase | 64 | 9 | Pjap03499<br>Pjap08406<br>Pjap31602<br>Pjap26690<br>Pjap12581<br>Pjap12354<br>Pjap29146<br>Pjap17933<br>Pjap29328 | 3E-55<br>3E-151<br>1E-35<br>0<br>4E-178<br>2E-177<br>1E-19<br>3E-31<br>3E-93 |
| DS,<br>Dammarenediol-II synthase | 33 | 6 | Pjap27407<br>Pjap14441<br>Pjap17978<br>Pjap16171<br>Pjap12813<br>Pjap32409 | 0<br>1E-143<br>1E-99<br>2E-81<br>2E-139<br>1E-105 |
| β-AS<br>β-amyrin synthase | 5 | 3 | Pjap27407<br>Pjap12813<br>Pjap02808 | 4E-70<br>0<br>1E-173 |
| β-A28O<br>β-amyrin 28-oxidase | 2 | 1 | Pjap11778 | 0 |
| D12H,<br>Dammarenediol 12-hydroxylase | 13 | 4 | Pjap05919<br>Pjap15083<br>Pjap12437<br>Pjap11778 | 2E-11<br>8E-136<br>4E-131<br>7E-38 |
| P6H,<br>Protopanaxadiol 6-hydroxylase | 9 | 3 | Pjap11778<br>Pjap12437<br>Pjap05919 | 2E-172<br>8E-06<br>4E-79 |

* Protein-coding genes of *P. notoginseng* were searched against the candidate unigenes of *P. ginseng* by using TBLASTX with a cutoff ≤ 1*E*-10.

**Table S5.** Identification of alternative splicing (AS) events in unigenes of *P. ginseng*.

| No | Cluster ID | Average length of ORFs analyzed (bp) | Functional description of clusters | No. of unigenes in cluster | No. of identified alternative splicing (AS) event | | Length of Insertion/Deletion (InDel) in AS (bp) | | | |
|---|---|---|---|---|---|---|---|---|---|---|
| | | | | | ≥ 100-bp | < 100-bp | #1 | #2 | #3 | #4 |
| 1 | KG_ISO_112454.162163_clust | 1669 | Protein TIME FOR COFFEE | 3 | 1 | 0 | 1,990 | | | |
| 2 | KG_ISO_090257.125297_clust | 1857 | DNA-directed RNA polymerase subunit beta | 2 | 1 | 0 | 2,105 | | | |
| 3 | KG_ISO_081709.109976_clust | 2267 | Isoleucine--tRNA ligase | 2 | 1 | 0 | 1,905 | | | |
| 4 | KG_ISO_016929.20726_clust | 1688 | Plant calmodulin-binding protein-related [TAIR10] | 2 | 1 | 0 | 1,803 | | | |
| 5 | KG_ISO_026079.31377_clust | 1699 | Putative K(+)-stimulated pyrophosphate-energized sodium pump (Fragment) | 3 | 1 | 0 | 1,118 | | | |
| 6 | KG_ISO_032384.38801_clust | 1340 | Eukaryotic translation initiation factor 3 subunit C | 2 | 1 | 0 | 1,191 | | | |
| 7 | KG_ISO_012760.15697_clust | 1712 | 5-methyltetrahydropteroyltriglutamate--homocysteine methyltransferase | 2 | 1 | 0 | 1,173 | | | |
| 8 | KG_ISO_007917.9737_clust | 1657 | cell wall protein AWA1-like isoform X1 [NCBI NR] | 5 | 1 | 0 | 951 | | | |
| 9 | KG_ISO_061372.79967_clust | 1076 | Hybrid signal transduction histidine kinase C | 3 | 1 | 0 | 755 | | | |
| 10 | KG_ISO_003330.4085_clust | 702 | Elongation factor Tu | 5 | 1 | 2 | 518 | 36 | 34 | |

| | | | | | | | | | | |
|---|---|---|---|---|---|---|---|---|---|---|
| 11 | KG_ISO_105641.150963_clust | 1003 | Pyrolysin | 3 | 1 | 0 | 674 | | | |
| 12 | KG_ISO_058574.76091_clust | 947 | F-box/LRR-repeat protein 15 | 2 | 1 | 0 | 843 | | | |
| 13 | KG_ISO_076296.100249_clust | 994 | ATP-dependent Clp protease ATP-binding subunit clpA homolog | 3 | 1 | 0 | 603 | | | |
| 14 | KG_ISO_103005.146715_clust | 759 | Zinc finger CCCH domain-containing protein 42 | 2 | 1 | 0 | 774 | | | |
| 15 | KG_ISO_070630.93214_clust | 994 | Copper-transporting P-type ATPase | 2 | 1 | 0 | 744 | | | |
| 16 | KG_ISO_025529.30721_clust | 606 | Protein MGA1 | 2 | 1 | 0 | 720 | | | |
| 17 | KG_ISO_077542.102329_clust | 986 | Protein EFR3 homolog cmp44E | 2 | 1 | 0 | 657 | | | |
| 18 | KG_ISO_012292.15123_clust | 807 | Putative heat shock 70 kDa protein 7 | 3 | 1 | 0 | 431 | | | |
| 19 | KG_ISO_052389.67114_clust | 858 | Translation initiation factor IF-2 | 2 | 1 | 0 | 558 | | | |
| 20 | KG_ISO_074970.98348_clust | 671 | heterotrimeric G-protein GTPase; nucleoside-triphosphatase [PMD] | 2 | 1 | 0 | 542 | | | |
| 21 | KG_ISO_089050.123153_clust | 847 | WD repeat-containing protein 1-A | 3 | 1 | 0 | 398 | | | |
| 22 | KG_ISO_080119.107003_clust | 807 | Cellulose synthase catalytic subunit [UDP-forming] | 4 | 1 | 0 | 339 | | | |
| 23 | KG_ISO_069305.91395_clust | 1058 | Cyclin-dependent kinase 20 | 3 | 1 | 0 | 378 | | | |
| 24 | KG_ISO_036247.44364_clust | 1077 | Interferon-induced GTP-binding protein Mx2 | 2 | 1 | 0 | 492 | | | |
| 25 | KG_ISO_075463.99111_clust | 749 | Glutamate synthase large subunit-like protein | 2 | 1 | 0 | 465 | | | |
| 26 | KG_ISO_038748.47910_clust | 1098 | Protein phosphatase 1 regulatory subunit 7 | 3 | 1 | 1 | 423 | 84 | | |

| | | | | | | | | | | |
|---|---|---|---|---|---|---|---|---|---|---|
| 27 | KG_ISO_032993.39683_clust | 878 | Probable methyltransferase PMT5 | 5 | 2 | 2 | 155 | 123 | 61 | 52 |
| 28 | KG_ISO_098598.139226_clust | 535 | UDP-galactose:fucoside alpha-3-galactosyltransferase | 6 | 1 | 1 | 215 | 46 | | |
| 29 | KG_ISO_053915.69278_clust | 763 | Meiotic PUF family protein 1 | 3 | 1 | 0 | 324 | | | |
| 30 | KG_ISO_129863.193408_clust | 564 | Probable eukaryotic translation initiation factor 4 gamma homolog | 8 | 1 | 1 | 235 | 12 | | |
| 31 | KG_ISO_063065.82449_clust | 758 | Elongation factor 4 | 6 | 2 | 0 | 282 | 198 | | |
| 32 | KG_ISO_001495.1817_clust | 1142 | Putative L-type lectin-domain containing receptor kinase I.1 | 3 | 1 | 0 | 304 | | | |
| 33 | KG_ISO_035051.42611_clust | 933 | Myosin light chain kinase, smooth muscle (Fragment) | 3 | 1 | 0 | 297 | | | |
| 34 | KG_ISO_073543.96209_clust | 709 | 26S protease regulatory subunit 7 homolog A | 3 | 1 | 0 | 297 | | | |
| 35 | KG_ISO_031533.37591_clust | 1624 | Peroxide stress-activated histidine kinase mak3 | 3 | 1 | 0 | 227 | | | |
| 36 | KG_ISO_030210.36169_clust | 654 | CUGBP Elav-like family member 1 | 4 | 1 | 1 | 291 | 72 | | |
| 37 | KG_ISO_023537.28432_clust | 542 | Mdo Malus domestica KNOTTED1-like homeobox gene 6 [PlantTFBS] | 2 | 1 | 0 | 376 | | | |
| 38 | KG_ISO_007420.9117_clust | 1163 | Sulfate adenylyltransferase subunit 1 | 2 | 1 | 0 | 375 | | | |
| 39 | KG_ISO_061261.79805_clust | 2789 | SED5-binding protein 3 | 3 | 1 | 0 | 273 | | | |
| 40 | KG_ISO_022867.27633_clust | 560 | Microtubule-associated protein 70-4 | 2 | 1 | 0 | 357 | | | |
| 41 | KG_ISO_001370.1670_clust | 1293 | Fasciclin-like arabinogalactan protein 18 | 3 | 1 | 0 | 261 | | | |
| 42 | KG_ISO_020333.24691_clust | 1554 | Inactive peptidyl-prolyl cis-trans isomerase shutdown | 2 | 1 | 0 | 348 | | | |

| | | | | | | | | | | |
|---|---|---|---|---|---|---|---|---|---|---|
| 43 | KG_ISO_036967.45360_clust | 1710 | Arachidonate 15-lipoxygenase | 3 | 1 | 1 | 309 | 99 | | |
| 44 | KG_ISO_131111.195656_clust | 375 | Probable methyltransferase PMT5 | 6 | 1 | 0 | 205 | | | |
| 45 | KG_ISO_000123.151_clust | 2232 | Sexual differentiation process putative subtilase-type proteinase isp6 | 3 | 1 | 0 | 252 | | | |
| 46 | KG_ISO_080714.108119_clust | 490 | B3 domain-containing protein Os02g0764100 | 12 | 1 | 1 | 184 | 79 | | |
| 47 | KG_ISO_026345.31690_clust | 1712 | Exocyst complex component 7 | 2 | 1 | 0 | 321 | | | |
| 48 | KG_ISO_012924.15896_clust | 779 | Solute carrier family 23 member 1 | 4 | 1 | 0 | 213 | | | |
| 49 | KG_ISO_038117.47021_clust | 577 | Dehydrin ERD14 | 3 | 1 | 0 | 240 | | | |
| 50 | KG_ISO_038576.47674_clust | 526 | Ubiquitin carboxyl-terminal hydrolase 33 | 3 | 2 | 0 | 264 | 207 | | |
| 51 | KG_ISO_048966.62197_clust | 705 | Growth-regulating factor 5 | 4 | 1 | 0 | 208 | | | |
| 52 | KG_ISO_033822.40850_clust | 398 | ATP-dependent rRNA helicase RRP3 | 3 | 1 | 0 | 227 | | | |
| 53 | KG_ISO_052026.66568_clust | 1812 | Holliday junction ATP-dependent DNA helicase RuvB | 2 | 1 | 0 | 300 | | | |
| 54 | KG_ISO_000634.796_clust | 1553 | Protein piccolo | 2 | 1 | 0 | 297 | | | |
| 55 | KG_ISO_109070.156713_clust | 558 | Beta-(1-->2)glucan export ATP-binding/permease protein NdvA | 2 | 1 | 0 | 295 | | | |
| 56 | KG_ISO_002789.3407_clust | 1511 | UBX domain-containing protein 4 | 3 | 1 | 0 | 219 | | | |
| 57 | KG_ISO_099730.141165_clust | 477 | Uncharacterized protein C4G9.04c | 4 | 1 | 0 | 184 | | | |
| 58 | KG_ISO_030520.36532_clust | 1305 | RNA-binding protein Nova-1 (Fragment) | 2 | 1 | 0 | 262 | | | |

| | | | | | | | | | | |
|---|---|---|---|---|---|---|---|---|---|---|
| 59 | KG_ISO_094048.131853_clust | 814 | Exocyst complex component 4 | 4 | 1 | 0 | 177 | | | |
| 60 | KG_ISO_054969.70864_clust | 718 | Homeobox protein knotted-1-like 8 (Fragment) | 10 | 1 | 0 | 147 | | | |
| 61 | KG_ISO_102488.145900_clust | 482 | Auxin-responsive protein IAA26 | 3 | 1 | 0 | 191 | | | |
| 62 | KG_ISO_091681.127724_clust | 741 | Nodal modulator 1 | 7 | 1 | 0 | 146 | | | |
| 63 | KG_ISO_070431.92941_clust | 488 | Next to BRCA1 gene 1 protein | 10 | 1 | 0 | 135 | | | |
| 64 | KG_ISO_055424.71483_clust | 2753 | Potassium-transporting ATPase B chain | 2 | 1 | 0 | 237 | | | |
| 65 | KG_ISO_021336.25847_clust | 498 | Unknown | 5 | 1 | 1 | 153 | 27 | | |
| 66 | KG_ISO_014023.17229_clust | 1042 | 4-alpha-glucanotransferase | 3 | 1 | 0 | 174 | | | |
| 67 | KG_ISO_063709.83358_clust | 1377 | F-box/LRR-repeat protein 7 | 4 | 1 | 0 | 153 | | | |
| 68 | KG_ISO_041754.52129_clust | 606 | AP-3 complex subunit delta | 5 | 1 | 0 | 143 | | | |
| 69 | KG_ISO_107936.154749_clust | 400 | Hsc70-interacting protein | 4 | 1 | 0 | 151 | | | |
| 70 | KG_ISO_112880.162869_clust | 1361 | Protein MPE1 | 3 | 1 | 0 | 168 | | | |
| 71 | KG_ISO_052743.67607_clust | 435 | Capsular polysaccharide biosynthesis glycosyltransferase CapM | 8 | 1 | 0 | 127 | | | |
| 72 | KG_ISO_000655.825_clust | 1187 | Probable protein phosphatase 2C 60 | 2 | 1 | 0 | 219 | | | |
| 73 | KG_ISO_064809.84926_clust | 1164 | Transcription factor TCP18 | 2 | 1 | 0 | 216 | | | |

| | | | | | | | | | | |
|---|---|---|---|---|---|---|---|---|---|---|
| 74 | KG_ISO_031617.37706_clust | 2931 | G-type lectin S-receptor-like serine/threonine-protein kinase At4g27290 | 2 | 1 | 0 | 216 | | | |
| 75 | KG_ISO_023305.28134_clust | 453 | Phosphate import ATP-binding protein PstB | 6 | 1 | 0 | 129 | | | |
| 76 | KG_ISO_012432.15296_clust | 912 | Chaperone protein DnaK | 3 | 1 | 0 | 158 | | | |
| 77 | KG_ISO_106352.152044_clust | 505 | Nuclear RNAi defective-3 protein | 8 | 1 | 0 | 120 | | | |
| 78 | KG_ISO_001291.1575_clust | 423 | Eukaryotic translation initiation factor 3 subunit A | 6 | 1 | 0 | 126 | | | |
| 79 | KG_ISO_121073.177494_clust | 657 | Probable salt tolerance-like protein At1g78600 | 3 | 1 | 0 | 156 | | | |
| 80 | KG_ISO_096467.135784_clust | 453 | Probable serine/threonine-protein kinase MARK-B | 4 | 1 | 0 | 202 | | | |
| 81 | KG_ISO_028625.34343_clust | 1285 | FRIGIDA-like protein 5 | 2 | 1 | 0 | 204 | | | |
| 82 | KG_ISO_034907.42427_clust | 2349 | Fibroblast growth factor receptor 1 | 2 | 1 | 0 | 204 | | | |
| 83 | KG_ISO_011306.13900_clust | 1359 | Uncharacterized membrane protein At1g16860 | 2 | 1 | 1 | 168 | 36 | | |
| 84 | KG_ISO_059249.77033_clust | 1422 | Protein unc-45 homolog A | 2 | 1 | 0 | 204 | | | |
| 85 | KG_ISO_028379.34049_clust | 551 | ABC transporter B family member 25, mitochondrial | 3 | 1 | 0 | 152 | | | |
| 86 | KG_ISO_032188.38532_clust | 2276 | Zinc finger CCCH domain-containing protein 38 | 3 | 1 | 1 | 171 | 39 | | |
| 87 | KG_ISO_015993.19649_clust | 950 | Probable protein kinase UbiB | 3 | 1 | 1 | 177 | 51 | | |
| 88 | KG_ISO_038833.48020_clust | 392 | Poly(A) polymerase | 6 | 1 | 0 | 121 | | | |

Table S6. Primer lists for qRT-PCR

| primer name | Forward | Reverse |
|---|---|---|
| ***Aux/IAA*** | | |
| *PgIAA7-a* | ATGAAAGGAACTGAAGCAATTGG | TCTCGTGTGCTTGTCATTGTTTG |
| *PgIAA7-b* | GATGCTCGTGGGCGATGT | GCAGGTTGCCGTTGATGTTT |
| *PgIAA8-a* | CTTGTTGGAGCAAATGGGTGTA | TCCAAGATGAAGGTCCGAGTGT |
| *PgIAA8-b* | TGGGTGCTTCAAGCCTGAAT | TGTCGTATTGAAGCCTTGATCAA |
| *PgIAA9-a* | GGCTGGATTGCGGATCAC | AATCGCACTTAACACTTCGGATTC |
| *PgIAA9-b* | CAGAAGGTTAAATGGTTTGCAGAA | GCACTTAACACTACGGATTCATATAAAGA |
| *PgIAA9-c* | CAAATGGGTGTAGGCACTGGAT | TGCTGCAGTATTCCAAGATGAAG |
| *PgIAA14* | TTGTTTGGATGCATGTGTCTCTT | TGACATAAAGGACACCAAATTAACAAC |
| *PgIAA16* | GGGAGATGTTGGTTGGTTCTTG | AGGCGACAACTAAGATCAAAGGA |
| *PgIAA18* | CGCAGTTGGAGTTGGTAGTAAGC | ACATCATTCCAAAATTCCTTTTCG |
| ***ARR*** | | |
| *PgARR4* | GGCGCCTTAAAATGACCAACT | GGGAATGACCCACCTATATTGTAAA |
| *PgARR6* | CGCCCTCGTTGCTAACTTCT | AACAGGGAATGACCCACCTATCT |
| *PgARR9a* | GAATAGAAAGAAGACCTAAAATGAAGGAA | CATCCATATCATGGTTTAATGCAAA |
| *PgARR9b* | AACCAGTGAGATTGTCAGATGTGAA | AGCCTTCCTCTTGTTATTACTGGATT |
| *PgARR15* | GCACCAATAATGCTAACAAGAGGAA | CAGTAGGAAAGATGGTAAGCCACAT |
